# Supplementary material for: Patterns of Intron Gain and Loss in Fungi
Source: PLoS Biol. 2004 Nov 30;2(12):e422. doi: 10.1371/journal.pbio.0020422 (PMC532390; doi:10.1371/journal.pbio.0020422)
Supplement: Table S1 — Also available at http://genes.mit.edu/NielsenEtAl/. (4.3 MB ZIP). [file pbio.0020422.st001.zip › NielsenEtAl/html/766.html]

AN5110.1.NCU01758.1.MG03159.1.FG05197.1


```
 CLUSTAL W (1.82) Multiple Sequence Alignments - Introns Inserted


Sequence 1: NCU01758.1	684 aa
Sequence 2: FG05197.1	643 aa
Sequence 3: MG03159.1	663 aa
Sequence 4: AN5110.1	584 aa
Alignment Length: 712 aa
Number Identitical Residues: 274 aa
Alignment Score (without introns) 14697


MG03159.1 	--------------------------MTDIRTSSPMPSAANQKGAVSAED----DGLTLA
NCU01758.1	MRSLQPAARCLALAQHLKSIPYANQPFSAFFSTTKHIMASNGTAIENGTHPLSSDGTTLS
FG05197.1 	--------------------------------MAAHISNGN-SALSPASG---YEGATLE
AN5110.1  	-------------------------------------MASQFSKADMGAG------LSLA
          	                                       .: .    .         :* 

MG03159.1 	DLPKSWRFTSALPADPEYPTPADSHKTPREQIGPRMVRGALFSWVRPERQRDPELLGVSP
NCU01758.1	ALPKSWHFTASLPPDAAFPTPADSHKADRDDLGPRQVKNAIFTWVRPEKQQDPELLAVSP
FG05197.1 	DLPKSWHFTESLPADSMFPTPADSHKTPRDQIGPRQVRNAAFTWVRPEEQKDPELLAVSP
AN5110.1  	ELPKSNVFTSKLPPDPAFDTPEASHKAQRERLYPRTVKGAAFTFVRPETTEDPELLGVSP
          	 ****  **  **.*. : **  ***: *: : ** *:.* *::****  .*****.***

MG03159.1 	AALRTLGIRPSEVHTDEFLQTAVGNKLHGWSEEKLEGDGYPWAQCYGG~FQFGQWANQLG
NCU01758.1	AAMRDLGLALSEADTEEFRQVAVGNKIIGWDEETLSGPGYPWAQCYGG~FQFGQWAGQLG
FG05197.1 	AALRDLGIKSGEETTENFKQMVAGNKLYGWDEEKLEG-GYPWAQCYGG~FQFGQWAGQLG
AN5110.1  	RAMKDLGLKPGEENTAQFKAVVAGNEFY-WDEE--NGGVYPWAQCYGV1VLAFRHEGTEA
          	 *:: **:  .*  * :*   ..**::  *.**  .*  ********  .   :  .  .

MG03159.1 	D--GRAISLFEATNPKTGERYEVQLKGAGLTPYSRFADGKAVLRSSIR~EFVASESLHAL
NCU01758.1	D--GRAISLFEGTNPATGVRYEVQLKGAGMTPYSRFADGKAVLRSSIR~EFIVSENLHAL
FG05197.1 	D--GRAISLFESTNPASGERHELQLKGAGLTPYSRFADGKAVLRSSIR~EFVVSEALNAL
AN5110.1  	EHQQRAISLFESTNPSTNVRYEVQLKGAGRTPYSRFADGKAVLRSSIH1VVAISNT----
          	: . *******.*** :. *:*:****** *****************:  .  *:     

MG03159.1 	GVPTTRALALSLLPHQKVRRETVEPGAIVVRFAQSWIR~LGTFDLLRARGDRDLIRKLAT
NCU01758.1	GIPSTRALAISLLPHSRVRRETMEPGAIVVRMAQSWLR~FGNFDILRARGDRKLVRQLAT
FG05197.1 	NIPTTRALSLTLLPDSKVRRERIEPGAIVLRFAQSWIR~LGNFDILRARGDRKLIRQLAT
AN5110.1  	------------------------------------CI2FGTFDLPHSRGDRNMVRKLAT
          	                                       :*.**: ::****.::*:***

MG03159.1 	YVAEDVLGGWENLPGRLVDPDKPSLEECSPALASMVESAAEDSSKSPIRRGIPEAEVEGP
NCU01758.1	YIGEEVFGGWDKLPGRLADPEGA-------------------PGDEPP-RGIPKETIEGP
FG05197.1 	YIAEDVFGGWEKLPGQLEDPDKP-------------------VDSPAPNRGVAADTIEGA
AN5110.1  	YVAEDVFNGWESLPGAVSVGKDQ-----------------QADSVEDPPRGLLGDKIQDH
          	*:.*:*:.**:.*** :   .                   .  .     **:    ::. 

MG03159.1 	SEMAENRFVRLYREICRRNAITVAHWQAYGFMNGVL~NTDNTSIIGLSMDYGPFAFVDVF
NCU01758.1	LGAEENRFHRLYREIIRRNALTVAKWQIYGFMNGVL~NTDNTSIMGLSIDFGPFAFMDNF
FG05197.1 	DGSEENRFTRFYREVVRRNAKVVAHWQAYGFMNGVL0NTDNTSIYGLSIDFGPFAFMDNF
AN5110.1  	HGVEENRFARLYREIARRNAKTVAAWQAYGFMNGVL~NTDNTSVYGLSLDYGPFAFMDNF
          	    **** *:***: **** .** ** ******** ******: ***:*:*****:* *

MG03159.1 	DPSYTPNHDDHALRYSYRNQPTIIWWNLVRLGEALGELLGAGADIDDPNFVDKGVK--DE
NCU01758.1	DPNYTPNHDDFALRYSYRNQATIIWWNLVRLGEALGELIGAGPEVDSESFVTNGLNFDDE
FG05197.1 	DPAYTPNHDDYALRYSYRNQPTIIWWNLVRFGEAIGELMGAGADVDHESFVTEGVKKEQE
AN5110.1  	DPQYTPNHDDHMLRYSYKNQPSVIWWNLVRLGECLGELIGAGPQVDDENFVKQGVT---E
          	** *******. *****:**.::*******:**.:***:***.::*  .** :*:.   *

MG03159.1 	AAAEPIISRAEALITQAGTEYRAVFLAEYKRLMCARLGLRTHKDSDFDKLFSELLDTMEA
NCU01758.1	AASKPIEERAHKLITQAGEEFKAVFMGEFKRLFTARLGLKTYKESDFDSLFDSLLNTMEA
FG05197.1 	EE---IVARAEKLITQAGEEYKAVFLAEYKRLMTTRLGLKTQKDKDFEVLFSEALDTMEA
AN5110.1  	DAAPALIKRAETIIERTGDEYRTVFLNQYKCLMSKRLGLRTQKESDFQELYSELLDTLEA
          	  : .:  **. :* ::* *:::**: ::* *:  ****:* *:.**: *:.. *:*:**

MG03159.1 	AELDFNFFFRRLSSLPLTDISTEADAEKNASLFFYE--EGVTGI-LGEEKARKRVGAWLC
NCU01758.1	LELDYNLFFRRLSTLKTADLQTEEARQKAAEVFFSQVEEVPGPG-TDKEKARKRVGEWLD
FG05197.1 	LELDFHHFFRRLSNVKLQDLATEEARKETASVFFHK--EGPPTI-TTEEDARARLAIWLE
AN5110.1  	LELDFNHFFRRLSSVPIVDLETEAGRKDIAHIFFHI--EGHGGIGYTEDSARERIAKWLD
          	 ***:: ******.:   *: **   :. * :**    *     .  ::.** *:. ** 

MG03159.1 	AWRDRVREDWG-------EPRDAERMEAMRRVNPN~FVPRGWILDEVIRRVEKEGERDVL
NCU01758.1	KWRVRIEEDWT-----TSAADSEERVAAMKRVNPS~FIPRGWILDEVIRRVEKQGERDVL
FG05197.1 	SWRERIIEDWKDEGDNVSDAKDLERIDAMKRVNPN~FVPRGWILDELIQRVEKDGERDVL
AN5110.1  	RWRQRVLEDWG-----TEADADIKRQEAMKKVNPK0FVPRGWILEEVIERVERRGDRDIL
          	 ** *: ***       .   . :*  **::***. *:******:*:*.***: *:**:*

MG03159.1 	RRLMHMSLNPFDESWNGKTFDG-----KIYEGDEAEEKRWIANVPKYKRAMQCSCSS
NCU01758.1	KRVLHMATHPFEDAWTGKEFEDGPTGKGVYQGDKAEEERWTGDVPQQKKAMQCSCSS
FG05197.1 	NRVMQMALNPFEDRWDGETFDG-----KTYTGNKDEELRWTDDPPQSERAMQCSCSS
AN5110.1  	GRVMNMALNPFNEEWG---------------LNREEEERFCGDVPRFQRAMMCSCSS
          	 *:::*: :**:: *                 :. ** *:  : *: ::** *****
```
